# Statin use and severe, critical, or fatal outcomes among individuals with coronavirus disease 2019

**DOI:** 10.1016/j.ijregi.2026.100946

**Published:** 2026-07-10

**Authors:** Adeel A. Butt, Peng Yan, Obaid S. Shaikh, Muzna I. Lone, Ahmed Demirci, Salih Akgun, Amyn A. Malik, Khurram Nasir

**Affiliations:** 1 VA Pittsburgh Healthcare System and Veterans Health Foundation, Pittsburgh, PA, USA; 2 Department of Medicine, Hackensack Meridian Health Network, JFK University Medical Center, Edison, NJ, USA; 3 Corporate Quality and Patient Safety Department, Hamad Medical Corporation, Doha, Qatar; 4 Department of Gastroenterology, Hepatology, and Nutrition, University of Pittsburgh Medical Center, Pittsburgh, PA, USA; 5 Peter O'Donnell Jr. School of Public Health, University of Texas Southwestern, Dallas, TX, USA; 6 Division of Cardiovascular Prevention and Wellness, Houston Methodist DeBakey Heart and Vascular Center, Houston, TX, USA; 7 Center for Cardiovascular Computational and Precision Health (C3-PH), Houston Methodist, Houston, TX, USA

**Keywords:** SARS-CoV-2, COVID-19, Statin, Outcomes, Hospitalization, Death

## Abstract

**Objectives:**

The role of statins in coronavirus disease 2019 (COVID-19) is controversial. We sought to determine the association between statin use and severe/critical/fatal disease in individuals with COVID-19.

**Methods:**

Using the US Department of Veterans Affairs COVID-19 database, we identified statin users with COVID-19 and inverse probability of treatment-weighted statin non-users. The primary outcome was severe, critical, or fatal disease within 28 days of diagnosis.

**Results:**

Among 8394 statin users and 59,412 inverse probability of treatment-weighted non-users, 277 statin users (3.3%) and 1493 non-users (2.5%) developed severe, critical, or fatal disease (absolute risk difference [ARD], −0.02; 95% confidence interval [CI], −0.19 to 0.16). Statin use was associated with more favorable outcomes among individuals aged >60 years (ARD, −0.79; 95% CI, −1.10 to −0.48), White individuals (ARD, −0.30; 95% CI, −0.51 to −0.09), and those with diabetes mellitus (ARD, −1.36; 95% CI, −1.82 to −0.90), coronary artery disease (ARD, −1.10; 95% CI, −1.89 to −0.31), stroke (ARD, −2.10; 95% CI, −3.65 to −0.55), chronic lung disease (ARD, −1.31; 95% CI, −1.94 to −0.68), chronic kidney disease (ARD, −2.46; 95% CI, −3.38 to −1.54), receipt of a booster vaccine dose (ARD, −0.34; 95% CI, −0.57 to −0.10), or no symptoms (ARD, −0.36; 95% CI, −0.59 to −0.14). After individuals with prevalent CVD were excluded, the nonsignificant association shifted toward favoring statin non-users (ARD, 0.62; 95% CI, 0.34-0.90).

**Conclusion:**

Statin users with COVID-19 did not experience a reduction in disease severity. In certain subgroups, favorable outcomes appeared to be related to the effects of statins on underlying disease processes.

## Highlights

• Statin use was not associated with reduced severe, critical, or fatal coronavirus disease 2019 outcomes within 28 days of the index diagnosis.
• A large national Veterans Affair cohort and inverse probability of treatment-weighted methodology were used to minimize confounding.
• Apparent subgroup benefits among individuals aged >60 years and those with diabetes or cardiovascular disease likely reflect statin effects on comorbidities.
• Excluding patients with cardiovascular disease shifted the association toward worse outcomes among statin users.
• The data do not support statins as adjunctive therapy for coronavirus disease 2019 outside established indications.

## Introduction

HMG-CoA reductase inhibitors, commonly referred to as “statins,” are recommended for primary prevention of cardiovascular disease (CVD) in adults aged 40-75 years who are at a moderate to high risk, defined by atherosclerotic cardiovascular disease risk score >10% [1,2]. Recent data suggest that over one-third of the US population report taking some type of statin [3]. While lowering of circulating lipids is the primary measurable target and indication for statin use, they have broad pleiotropic effects on endothelial function with significant anti-inflammatory, anti-oxidant, antithrombotic, and immunomodulatory properties that extend well beyond lipid regulation [[4], [5], [6], [7]]. These properties have generated interest in their potential role in infectious and inflammatory conditions. In particular, statin use has been associated with improved virologic outcomes in hepatitis C virus infection and with attenuation of inflammatory responses in respiratory illness, including possible reduced severity and mortality in community-acquired pneumonia and influenza [[8], [9], [10]].

Based upon their pleiotropic effects and some anecdotal reports, several studies have evaluated the effect of statins in individuals with coronavirus disease 2019 (COVID-19). While there are some differences in study design and population in these reports, there is wide variation in results. Some observational studies have demonstrated a beneficial effect on survival [[11], [12], [13]], while at least one large international randomized controlled trial in critically ill patients [14] and one meta-analysis [15] failed to show a beneficial effect. Another retrospective study in hospitalized patients found an increased risk of mechanical ventilation requirement, and intensive care unit admissions, and mortality among those who received statins [16].

COVID-19 has transitioned from an acute global pandemic to an endemic phase characterized by persistent viral circulation, recurrent waves of infection, and continued morbidity and mortality among high-risk individuals [17,18]. Despite this transition, the burden of severe disease in vulnerable populations, particularly those with underlying comorbidities, underscores the continued importance of identifying pharmacologic strategies that may mitigate disease severity. This study aims to elucidate the association between statin use and the development of severe, critical, or fatal COVID-19 outcomes within 28 days of index diagnosis in a large, national cohort. To achieve this, we employed a robust methodological approach designed to minimize confounding and selection bias.

## Methods

### Study setting and participants

The study used the US Department of Veterans Affairs (VA) COVID-19 database, which contains detailed demographic, clinical, laboratory, vital-status, and episode-of-care information for all veterans with laboratory-confirmed COVID-19, including COVID-19 vaccination within the VA health care system. Tests and vaccinations received outside the VA are captured through self-report or insurance claims data. The database is updated in real time with information from multiple validated sources.

We identified all individuals with at least one positive severe acute respiratory syndrome coronavirus 2 (SARS-CoV-2) polymerase chain reaction (PCR) test and at least two clinical encounters at any VA health care facility after January 1, 2019. We excluded individuals with a missing body mass index or testing location, any hospitalization within 30 days before the index positive SARS-CoV-2 PCR test, or a diagnosis of human immunodeficiency virus infection, organ transplantation, or any cancer (except nonmelanoma skin cancer) after January 1, 2017, because of their inherently elevated risk of complications related to disease complexity, immunosuppressive or chemotherapeutic treatment, and altered life expectancy. Among eligible individuals, the exposed group comprised those who received at least one prescription for an approved statin within ±7 days of the index positive test. The unexposed group comprised individuals with no statin prescription from 365 days before through 28 days after the index test; this wider window was intended to exclude residual or intermittent statin use.

Our primary analysis used an inverse probability of treatment weights (IPTW) to address potential imbalance in patient characteristics and statin prescription between cases (statin users or the exposed group) and controls (statin non-users or the unexposed group), establishing a pseudo-population in which exposure is independent of measured covariates. IPTW was calculated conditional on age, race, sex, body mass index, month of COVID-19 diagnosis, hypertension, diabetes, coronary artery disease, stroke, chronic kidney disease, chronic obstructive pulmonary disease, presence of COVID-19 symptoms at presentation, vaccination status at index date (unvaccinated/single dose vs two or more doses), and geographic location of diagnosis. Stabilized weights were used, and propensity scores below the 2.5^th^ percentile among statin users or above the 97.5^th^ percentile among statin non-users were excluded to minimize the disproportionate influence of extreme weights [19,20].

### Outcome

The primary outcome was development of severe, critical or fatal disease within 28 days of the index COVID-19 diagnosis, defined as admission to an intensive care unit, mechanical ventilation, or death within that period. We have used this same outcome definition in numerous previous publications [21,22].

### Statistical analysis

Baseline characteristics were compared between statin users and statin non-users before and after IPTW. Standardized mean differences (SMD) were used to assess adequacy of matching, where an SMD <0.1 indicates good matching. Absolute risk differences (ARDs) with corresponding 95% confidence intervals (CIs) were calculated before and after applying IPTW, where values above zero favored statin non-users, and a value below zero favored statin users. Hazard ratios with CIs were estimated utilizing a weighted Cox proportional hazards model for the IPTW-adjusted cohort and a standard Cox model for the unweighted cohort; the same models were used to generate Kaplan-Meier curves and at-risk/event counts. Subgroups analyses were performed using ARDs and Kaplan-Meir curves after stratification by demographic and clinical characteristics.

### Additional analyses

To assess reproducibility and robustness of our results, we repeated all analyses using propensity score matching. Each statin-user was matched 1:1, without replacement, to a control with no statin prescription in the prior 365 days through 28 days after diagnosis, using a greedy-matching algorithm with a caliper width of 0.1 SDs. Matching variables included age, race, sex, body mass index, month of COVID-19 diagnosis, comorbidities (hypertension, diabetes, coronary artery disease, stroke, chronic kidney disease stage (1-2, 3, 4-5), chronic obstructive pulmonary disease), COVID-19 symptoms at presentation (present/absent), vaccination status at index COVID-19 date (unvaccinated or single dose; two or more doses), and geographic location of diagnosis.

Since a key clinical indication for statin use is secondary prevention of cardiovascular disease, we stratified the cohort by baseline cardiovascular disease status and repeated the primary analysis after excluding individuals with prevalent cardiovascular disease, in order to isolate the effect of statin use among individuals less likely to be receiving statins specifically for established cardiovascular indications.

### Ethical approval

The study was reviewed by the Institutional Review Board at the VA Pittsburgh Healthcare System (Study Number Pro00003536) and determined to be exempt from Institutional Review Board review. Since there was no contact with any of the participants, and due to its exempt status, the informed consent requirement was not applicable.

## Results

**Figure 1:**
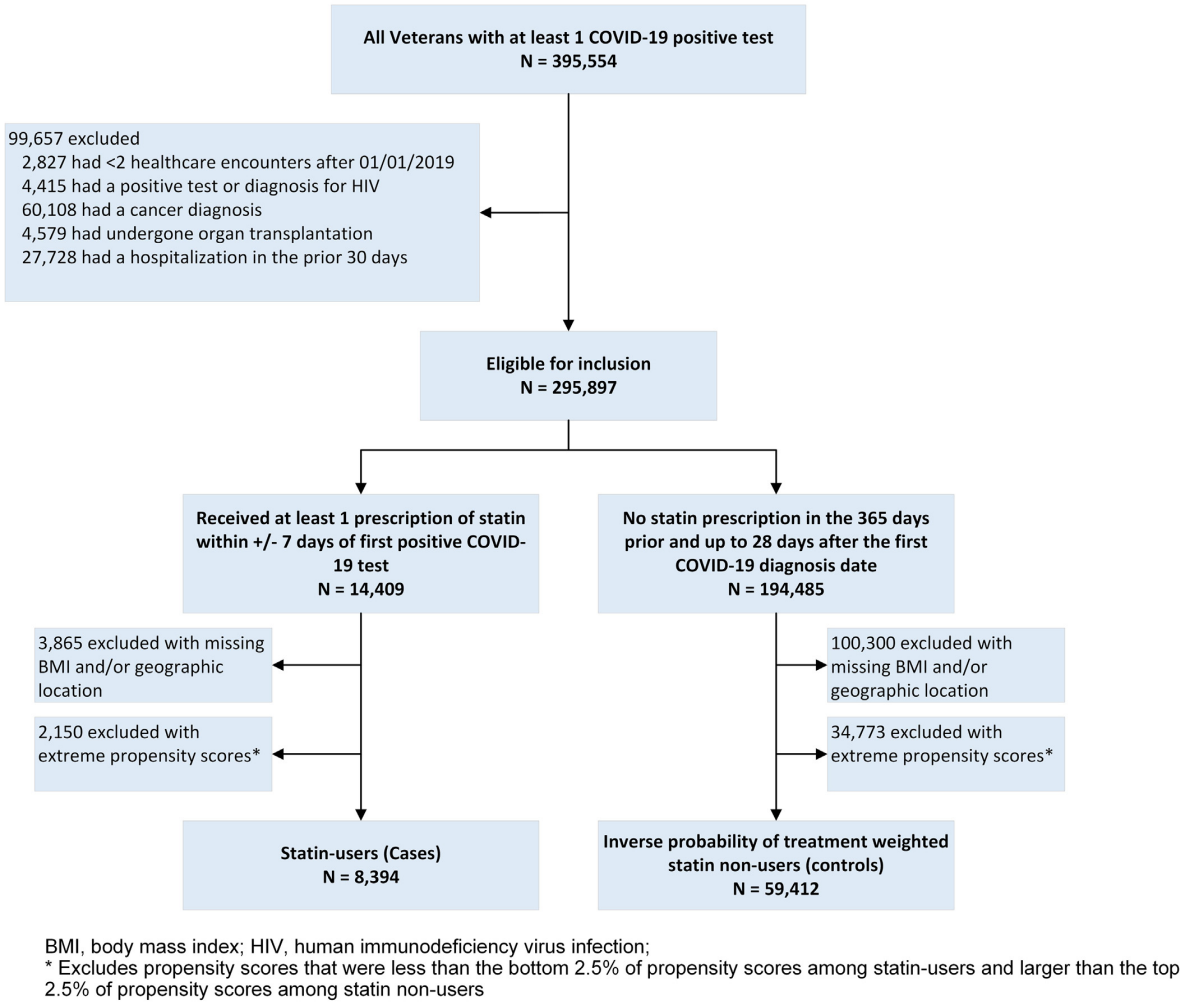
Cohort construction.

Abbreviations: BMI, body mass index; COVID-19, coronavirus disease 2019; HIV, human immunodeficiency virus.

A total of 395,554 individuals with confirmed COVID-19 were identified, of whom 295,897 were eligible for inclusion (Figure 1). Among eligible individuals, 14,409 had received at least one statin prescription within ±7 days of the index COVID-19 diagnosis, whereas 194,485 had no statin prescription from 365 days before through 28 days after the index diagnosis. In the final analysis, 8394 statin users were compared with 59,412 statin non-users using the IPTW method described above (Figure 1).

**Figure 2:**
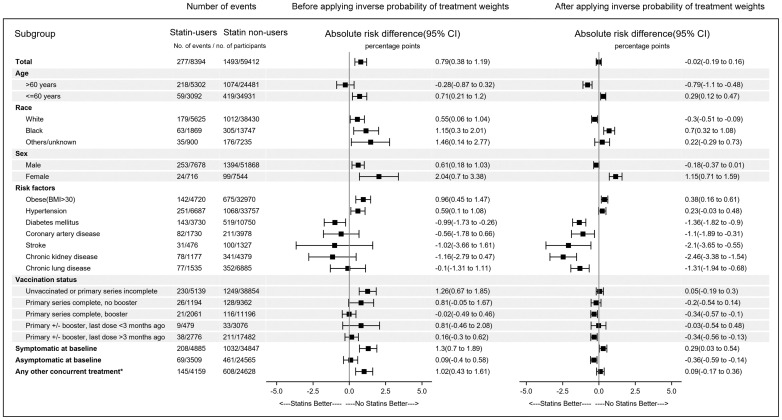
Absolute risk difference in outcomes among statin users and statin non-users. Primary outcome event was development of severe, critical or fatal disease within 28 days of the index COVID-19 diagnosis.

Abbreviations: BMI, body mass index; CI, confidence interval; COVID-19, coronavirus disease 2019.

Baseline characteristics of the two groups before and after IPTW are provided in Table 1. Among statin users, the median age was 61 years, 67% were White, 88% were male, 54% were obese, the median body mass index was 30.4 kg/m^2^, 67% were unvaccinated or had not completed a primary series, and 57% were symptomatic at presentation. Among statin non-users, the median age was 58 years, 65% were White, 88% were male, 55% were obese, the median body mass index was 30.6 kg/m^2^, 65% were unvaccinated or had not completed a primary series, and 58% were symptomatic at presentation (Table 1). There were 277 events among statin users (277/8394; 3.3%) and 1493 events among statin non-users (1493/59,412; 2.5%), corresponding to an ARD of −0.02 (95% CI, −0.19 to 0.16), which suggested no significant association between statin use and the outcome (Figure 2). Subgroup analyses showed more favorable outcomes among statin users aged >60 years, White individuals, and those with diabetes mellitus, coronary artery disease, stroke, chronic lung disease, or chronic kidney disease. A beneficial association was also observed among vaccinated individuals who had received a booster dose and those who were asymptomatic at baseline. ARDs suggested worse outcomes among individuals aged <60 years, Black individuals, women, individuals with obesity, and those who were symptomatic at baseline (Figure 2).

**Table 1:** Baseline characteristics of statin users and statin non-users before and after inverse probability of treatment weighting.

	Before IPTW			After IPTW		
	Statin users	Statin non-users	*P* value	Statin users	Statin non-users	SMD
	N = 8394	N = 59,412		N = 8394	N = 59,412	
**Demographics**						
Age, years (mean or median, SD or IQR)	65 (56-72)	57 (47-68)	<0.0001	61 (52-70)	58 (48-69)	0.0918
Race, %			<0.0001			0.0438
White	67.01%	64.68%		66.93%	65.02%	
Black	22.27%	23.14%		21.52%	22.98%	
Other/unknown	10.72%	12.18%		11.54%	12.00%	
Sex, % male	91.47%	87.30%	<0.0001	88.30%	87.84%	0.0142
**Risk factors, %**						
Median body mass index. kg/m^2^ (IQR)	30.7 (27-34.9)	30.6 (27-34.9)	0.10	30.4 (27-34.4)	30.6 (27-35)	-0.0144
Obese (% with BMI ≥ 30 kg/m^2^	56.23%	55.49%	0.2035	54.41%	55.47%	-0.0215
Hypertension	79.66%	56.82%	<0.0001	60.73%	59.69%	0.0212
Diabetes mellitus	44.44%	18.09%	<0.0001	23.67%	21.50%	0.0519
Coronary artery disease	20.61%	6.70%	<0.0001	9.45%	8.52%	0.0323
Stroke	5.67%	2.23%	<0.0001	3.55%	2.71%	0.0481
Chronic kidney disease	14.02%	7.37%	<0.0001	9.63%	8.28%	0.0471
Chronic obstructive pulmonary disease	18.29%	11.59%	<0.0001	14.02%	12.49%	0.0454
**Other therapies, %**						
Systemic corticosteroids	45.13%	37.59%	<0.0001	43.15%	37.91%	0.107
Remdesivir	0.00%	0.01%	0.36	0.00%	0.01%	-0.0155
Nirmatrelvir or molnupiravir	10.07%	7.95%	<0.0001	8.78%	8.11%	0.0239
**COVID-19 related**						
Symptomatic disease, %	58.20%	58.65%	0.43	56.61%	58.54%	-0.0391
Vaccination status, %			<0.0001			0.0857
Unvaccinated or primary series incomplete	61.22%	65.40%		66.96%	64.89%	
Primary series complete	14.22%	15.76%		13.32%	15.63%	
Primary series plus ≥1 booster	24.55%	18.84%		19.72%	19.48%	

Abbreviations: BMI, body mass index; COVID-19, coronavirus disease 2019; IQR, interquartile range; IPTW, inverse probability of treatment weighting; SMD, standardized mean difference.

**Figure 3:**
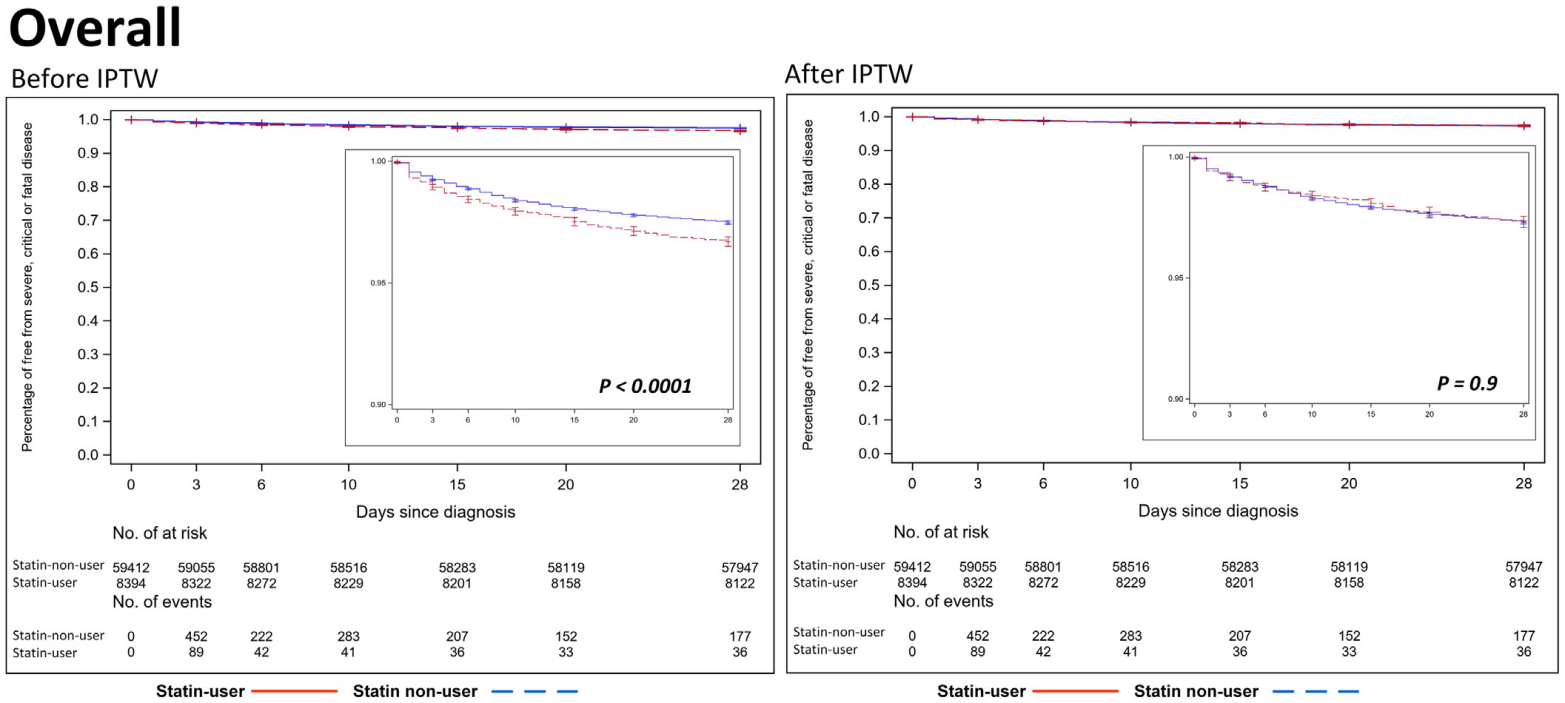
Kaplan-Meier curves showing the proportion of individuals who did not develop severe, critical, or fatal disease within 28 days of the index COVID-19 diagnosis among statin users and statin non-users.

Abbreviations: COVID-19, coronavirus disease 2019; IPTW, inverse probability of treatment weighting.

Kaplan-Meier curves demonstrated worse outcomes among statin users overall before IPTW, an association that was not observed after IPTW (Figure 3). In subgroup comparisons after IPTW, statin users aged >60 years and those with diabetes or chronic kidney disease were less likely to experience a severe, critical, or fatal outcome (Supplementary Figure 1). Complete comparisons before and after IPTW are provided in Supplementary Figure 2, panels A-I. Other than in the aforementioned subgroups, significant associations with the primary outcome among statin users before IPTW were no longer evident after IPTW.

### Additional analyses

We repeated all analyses in propensity score-matched groups from the entire cohort. A total of 8394 matched pairs were analyzed. A modest overall benefit was observed among statin users (ARD, −0.61; 95% CI, −1.17 to −0.04; Supplementary Figure 3). Other associations were generally in the same direction as those in the primary analysis. Statin users were less likely to experience a negative outcome if they were aged >60 years, male, had diabetes or chronic kidney disease, had completed vaccination with or without a booster, or were asymptomatic at presentation (Supplementary Figure 3).

**Figure 4:**
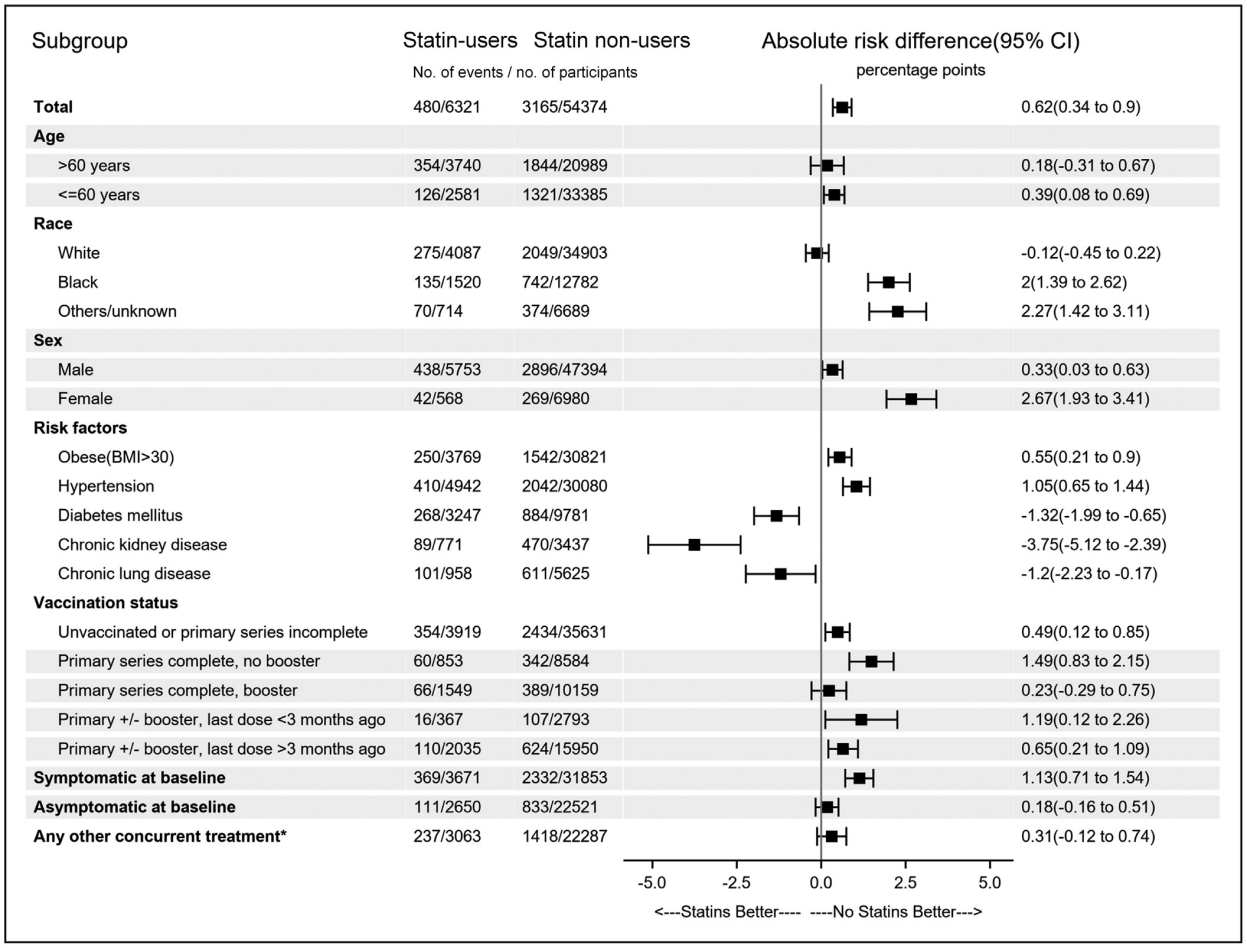
Absolute risk difference in outcomes among statin users and statin non-users after excluding individuals with a cardiovascular disease (coronary artery disease and stroke) diagnosis at baseline. Primary outcome event was development of severe, critical or fatal disease within 28 days of the index COVID-19 diagnosis. Abbreviations: CI, confidence interval; COVID-19, coronavirus disease 2019.

Since statins are primarily used for risk reduction in individuals with cardiovascular disease, we repeated our analysis after excluding individuals with cardiovascular disease (coronary artery disease and stroke). This analysis included 6321 statin users and 54,374 statin non-users in the IPTW analysis after excluding 7111 individuals with cardiovascular disease at baseline. Statin users experienced worse outcomes (ARD 0.62, 95% CI 0.34-0.90) for the primary outcome compared with statin non-users (Figure 4).

To address potential bias introduced by multiple hypothesis testing, all primary analyses were repeated using Bonferroni-adjusted estimates and CIs (Supplementary Figures 4 and 5). There were no appreciable changes in the direction or magnitude of the associations compared with the primary analysis.

## Discussion

In this large real-world study of the association between statin use and short-term disease severity in individuals with COVID-19, we found no evidence that statin use reduced progression to severe, critical, or fatal disease within 28 days of the index diagnosis. Importantly, these findings are limited to the acute disease period and do not preclude possible long-term benefits of statin use in individuals with COVID-19. Given the well-documented association between SARS-CoV-2 infection and increased long-term risk of cardiovascular events, including myocardial infarction and ischemic stroke, the potential role of statins in mitigating these sequelae warrants dedicated investigation beyond the short-term period examined here.

The primary role of statins in clinical practice is prevention of cardiovascular disease events and all-cause mortality through lipid lowering, with additional contributions from their anti-inflammatory, antithrombotic, and endothelial stabilizing properties [1,23]. These pleiotropic effects have generated considerable interest in their potential utility in COVID-19, particularly given autopsy findings of severe endothelial and microvascular injury, widespread thrombosis, and vascular angiogenesis in the lungs of individuals who died from the disease [[24], [25], [26], [27], [28], [29]]. Despite this mechanistic rationale, the role of statins in modifying COVID-19 disease severity has remained controversial. While several observational studies have reported beneficial associations with survival [[11], [12], [13]], a large international randomized controlled trial in critically ill patients and a meta-analysis of randomized trials failed to demonstrate benefit [14,15], and at least one retrospective study reported increased rates of mechanical ventilation, intensive care unit admission, and mortality among statin recipients [16]. Our findings are consistent with the null results of the randomized evidence and underscore the distinction between statins’ established cardiovascular protective effects and any putative direct effect on COVID-19 disease progression.

Our findings indicate no overall short-term benefit of statin use in preventing progression to severe, critical, or fatal disease in individuals with COVID-19. Certain subgroups, particularly individuals aged >60 years and those with pre-existing diabetes or chronic kidney disease, had more favorable outcomes with statin use; however, the shift in associations from unweighted to weighted analyses suggests that these apparent benefits were largely driven by underlying patient characteristics rather than a direct effect on COVID-19 or SARS-CoV-2. This interpretation is further supported by the observation that excluding individuals with prevalent cardiovascular disease shifted the risk difference toward favoring statin nonuse, implying that statins exert their influence primarily by modifying the underlying cardiovascular risk profile. Although the pleiotropic effects of statins may help attenuate disease severity in selected populations, further studies are needed to clarify these associations.

Strengths of our study include a large and diverse national population and a validated, robust database with extensive baseline and longitudinal information. Several limitations inherent to retrospective and observational studies should be considered when interpreting our findings. These include non-systematic data collection, non-randomized treatment decisions, variable disease severity at presentation, and the possibility of residual confounding from unmeasured variables, including smoking history, frailty, socioeconomic status, baseline lipid levels, and healthcare utilization intensity which could not be incorporated into our propensity model. Although IPTW was employed to balance measured covariates, corticosteroid use remained just above ideal balance after weighting with an SMD of 0.107 which should ideally be <0.1 but can be acceptable from 0.1 to 0.25 [30]; however, its potential influence on outcomes cannot be fully excluded. Our study population consisted entirely of Veterans receiving care at VA facilities nationwide and may not be fully representative of the broader US population; however, veterans in care tend to be older, more racially and ethnically diverse, and carry a higher comorbidity burden than the general population, allowing more robust examination of these factors [31]. The VA database captures all PCR-confirmed diagnoses within the system, and individuals were identified at their first confirmed positive test; however, prior infections occurring outside the VA system cannot be definitively excluded, which may affect the assumption of index infection status. Furthermore, the study period spans multiple SARS-CoV-2 variant eras, including Ancestral, Delta, and Omicron predominant periods, each associated with meaningfully different disease severity profiles and treatment landscapes; while month of diagnosis was included as a covariate in the propensity model, this may not fully account for the differential impact of distinct variant eras on outcomes. Finally, and importantly, this study evaluated only short-term outcomes within 28 days of index COVID-19 diagnosis and does not address the potential long-term benefits of statin use. In particular, given the well-documented association between SARS-CoV-2 infection and increased long-term risk of adverse cardiovascular events, including myocardial infarction and ischemic stroke, the impact of statin use on these sequelae represents an important and unaddressed question that warrants dedicated future investigation.

In conclusion, in this large national cohort, statin use was not associated with a reduction in short-term disease severity among individuals with COVID-19 within 28 days of index diagnosis. In certain subgroups, particularly older individuals and those with pre-existing diabetes, chronic kidney disease, or cardiovascular disease, more favorable outcomes were observed among statin users; however, these associations appear to reflect the established protective effects of statins on underlying comorbid conditions rather than any direct effect on COVID-19. Our findings pertain specifically to the adjunctive use of statins as a therapeutic intervention for COVID-19 and should not be interpreted as evidence against statin use for their established indications. Whether statin use confers long-term benefits in the context of COVID-19, particularly with respect to the cardiovascular sequelae, warrants further investigation.

## CRediT authorship contribution statement

**Adeel A. Butt:** Conceptualization, Formal analysis, Investigation, Methodology, Project administration, Supervision, Writing – original draft, Writing – review & editing, Validation. **Peng Yan:** Data curation, Formal analysis, Software, Validation, Writing – review & editing. **Obaid S. Shaikh:** Writing – review & editing. **Muzna I. Lone:** Writing – review & editing. **Ahmed Demirci:** Writing – review & editing. **Salih Akgun:** Writing – review & editing. **Amyn A. Malik:** Writing – review & editing. **Khurram Nasir:** Writing – review & editing.

## Declaration of competing interest

Dr. Butt has received investigator-initiated grant funding from Gilead Sciences and Merck & Co. to the Veterans Health Foundation of Pittsburgh; this funding is unrelated to the work presented here. Dr. Butt has served as a consultant to Pfizer Inc. Dr. Butt is also supported by the National Institute of Allergy and Infectious Diseases of the National Institutes of Health under Award R21AI174041 (principal investigator: Butt). The content is solely the responsibility of the authors and does not necessarily represent the official views of the Department of Veterans Affairs, the Veterans Health Foundation of Pittsburgh, or the National Institutes of Health.

## Appendix Supplementary materials

Image, application 1
