# Supplementary material for: Statin use and severe, critical, or fatal outcomes among individuals with coronavirus disease 2019
Source: IJID Reg. 2026 Jul 10;20:100946. doi: 10.1016/j.ijregi.2026.100946 (PMC13446357; doi:10.1016/j.ijregi.2026.100946)
Supplement: Supplementary file 1 [file mmc1.docx]

**Supplementary Data**

Supplementary figure 1. Kaplan-Meier curves depicting proportion of individuals without development of severe, critical or fatal disease within 28 days of the index COVID-19 diagnosis among statin-users and stain non-users for the subgroups that demonstrated a significant association.

Supplementary figure 2. Kaplan-Meier curves demonstrating the proportion of individuals free of severe, critical or fatal disease (i.e. absence of care requirement in the intensive care unit, mechanical ventilation or death) within 28 days of the index COVID-19 diagnosis. The graphs demonstrate the results in demographic and clinical subgroups before and after applying inverse probability of treatment weights.

**A. By age group.**

**B. By body mass index.**

**C. By presence of diabetes at baseline.**

**D. By presence of coronary artery disease at baseline.**

**E. By presence of stroke at baseline.**

**F. By presence of chronic kidney disease at baseline.**

**G. By receipt of other treatments for COVID-19.**

**H. By presence of symptoms at time of presentation.**

**I. By vaccination status.**

Supplementary figure 3. Absolute risk difference in development of severe, critical or fatal disease within 28 days of the index COVID-19 diagnosis among statin-users and statin non-users comparing results from analysis using the unmatched groups, and after propensity score matching and inverse probability of treatment weighting.

Supplementary figure 4. Absolute risk difference in development of severe, critical or fatal disease within 28 days of the index COVID-19 diagnosis among statin-users and statin non-users after applying Bonferoni correction for multiple comparisons.

Supplementary figure 5. Absolute risk difference in development of severe, critical or fatal disease within 28 days of the index COVID-19 diagnosis among statin-users and statin non-users after applying Bonferoni correction for multiple comparisons and excluding those with a diagnosis of cardiovascular disease event.
